# Potential inhibitors for blocking the interaction of the coronavirus SARS-CoV-2 spike protein and its host cell receptor ACE2

**DOI:** 10.1186/s12967-022-03501-9

**Published:** 2022-07-14

**Authors:** Changzhi Li, Hongjuan Zhou, Lingling Guo, Dehuan Xie, Huiping He, Hong Zhang, Yixiu Liu, Lixia Peng, Lisheng Zheng, Wenhua Lu, Yan Mei, Zhijie Liu, Jie Huang, Mingdian Wang, Ditian Shu, Liuyan Ding, Yanhong Lang, Feifei Luo, Jing Wang, Bijun Huang, Peng Huang, Song Gao, Jindong Chen, Chao-Nan Qian

**Affiliations:** 1 State Key Laboratory of Oncology in South China and Collaborative Innovation Center for Cancer Medicine, Sun Yat-Sen University Cancer Center, Guangzhou, 510060 China; 2 Department of Nasopharyngeal Carcinoma, Sun Yat-Sen University Cancer Center, Guangzhou, 510060 China; 3 Exploring Health, LLC., Guangzhou, 510663 China; 4 Department of Gynecology, Guangzhou Women and Children’s Medical Center, Guangzhou Medical University, Guangzhou, 510623 China; 5 Guangzhou Regenerative Medicine and Health Guangdong Laboratory, Guangzhou, 510530 China; 6 Guangzhou Concord Cancer Center, Guangzhou, 510555 China

**Keywords:** Coronavirus, SARS-CoV-2, COVID-19, Spike protein, ACE2, Tannic acid, 9-Methoxycanthin-6-one

## Abstract

**Background:**

The outbreak of SARS-CoV-2 continues to pose a serious threat to human health and social. The ongoing pandemic of COVID-19 caused by the severe acute respiratory syndrome coronavirus 2 (SARS-CoV-2) has made a serious threat to public health and economic stability worldwide. Given the urgency of the situation, researchers are attempting to repurpose existing drugs for treating COVID-19.

**Methods:**

We first established an anti-coronavirus drug screening platform based on the Homogeneous Time Resolved Fluorescence (HTRF) technology and the interaction between the coronavirus spike protein and its host receptor ACE2. Two compound libraries of 2,864 molecules were screened with this platform. Selected candidate compounds were validated by SARS-CoV-2_S pseudotyped lentivirus and ACE2-overexpressing cell system. Molecular docking was used to analyze the interaction between S protein and compounds.

**Results:**

We identified three potential anti-coronavirus compounds: tannic acid (TA), TS-1276 (anthraquinone), and TS-984 (9-Methoxycanthin-6-one). Our in vitro validation experiments indicated that TS-984 strongly inhibits the interaction of the coronavirus S protein and the human cell ACE2 receptor. Additionally, tannic acid showed moderate inhibitory effect on the interaction of S protein and ACE2.

**Conclusion:**

This platform is a rapid, sensitive, specific, and high throughput system, and available for screening large compound libraries. TS-984 is a potent blocker of the interaction between the S-protein and ACE2, which might have the potential to be developed into an effective anti-coronavirus drug.

**Supplementary Information:**

The online version contains supplementary material available at 10.1186/s12967-022-03501-9.

## Background

Coronavirus disease 2019 (COVID-19) is caused by a novel positive-sense, single-stranded RNA coronavirus, named severe acute respiratory syndrome coronavirus 2 (SARS-CoV-2) [1]. To date, SARS-CoV-2 has infected approximately 530 million people and caused more than six million deaths worldwide, and it continues to pose a serious threat to human health as well as social and economic stability, thus calling for the development of highly effective therapeutics and prophylactics. Even though several drugs and vaccines have been developed and approved for emergency use in some countries, there are few specific nor highly effective anti-SARS-CoV-2 drugs available.

## Methods

### Compound library and candidate compounds

TA was identified from the Food and Drug Administration (FDA) compound library (US Drug Collection), a unique collection of 1280 small molecules that have reached clinical trials. TA was purchased from the United States of America. TS-984 and TS-1276 from the Topscience compound library (Cat. No. L6000, Topscience, Shanghai, China), and contained 1584 natural compound products. All the compounds were provided in 10 mM of dimethyl sulfoxide (DMSO). The compounds of the libraries were diluted to 100 μM for HTRF assay.

### Experimental cell lines and reagents

Cell lines: ACE2-overexpressing 293T cells (with mCherry labeled vector), 293T cells (only vector overexpressed), ACE2-overexpressing Capan2 cells (mCherry labeled vector), Capan2 cells (only vector overexpressed), were all donated from Chaonan Qian’s laboratory (SYSUCC, Guangzhou, China). SARS-CoV-2_S (D614G)-pseudotyped lentivirus (> 10^8^ TU/ml, 10 × 100 μl, HBSS buffer solution) vector was VB900088-2229upx, with the polybrene (5 mg/ml) both of which were purchased from VectorBuilder China (Guangzhou, China).

ACE2 tagged with C-Fc and labeled with DRA36, and 2019-nCoV-S-protein tagged with C-6His and labeled with C05Y, were purchased from Novoprotein (Shanghai, China). Positive inhibitor control Nafamostat mesylate (T2392) and Emodin (T2869) were purchased from Topscience (Shanghai, China). The PAb Anti Human IgG-d2 (61HFCDAB) and MAb Anti-6HIS-Tb cryptate Gold (61HI2TLB) were purchased from CisBio Bioassays (Codolet, France). DMEM (Gibco, Cat#C11995500BT), FBS (Invitrogen, Cat# 10500064), 0.25% Trypsin (Invitrogen, Cat#25200056). D-PBS (Invitrogen, Cat#14190169).

### Preparation of the working compound library

To prepare the working compound library, 2 μl of each compound from the stock library was dispensed into each well of the 384-well plate using a Voyager pipette (Integra, Zizers, Switzerland). A phosphate buffered saline (PBS) (1×) was used as a negative control, and emodin (100 μM) was used as a positive control [2, 3]. The final reaction volume was 20 μl, and the final compound concentration was 100 μM in PBS.

### Optimization of the HTRF-based spike protein/ACE2 inhibitor screening system

To optimize the interaction between the spike protein (S protein) and ACE2, we performed cross-titration experiments to determine the maximal effect. In brief, ACE2 and SARS-Cov-2 S-protein were prepared at multiple concentrations with a PBS containing 0.1% BSA, 5 μl ACE2 (Mammalian, C-Fc, DRA36, Novoprotein) and 5 μl SARS-Cov-2 S-protein RBD (Mammalian, C-6His, C05Y, Novoprotein). Each concentration was added into each well of the 384-microplate (ProxiPlate™ 384-shallow well Microplates, 66PLP96025, CISBIO) and the mixture incubated at 37 °C for 1 h. Next, 5 μl PAb Anti Human IgG-d2 (61HFCDAB) and 5 μl MAb Anti-6HIS-Tb cryptate Gold (61HI2TLB) were added to each well with the ACE2/S-protein RBD mixture (final reactive volume of 20 μl) following the supplier’s protocols. After 30 min final incubation at room temperature, HTRF signals were measured using a Multimode Reader (Spark 10M, Tecan) equipped with an excitation filter of 340 nm, and fluorescence detected at 620 and 665 nm with a lag time of 100 μs and an integration time of 200 μs. The results were analyzed using a two-wavelength signal ratio: [intensity (665 nm)/intensity (620 nm)] × 10^4^ (HTRF Ratio). The Z factor was calculated using the following equation:

$${\text{Z}} = 1 - \frac{{3*{\text{SD}}\;{\text{negative}} + 3*{\text{SD}}\;{\text{positive}}}}{{{\text{MEAN}}\;{\text{negative}} - {\text{MEAN}}\;{\text{positive}}}}$$

Standard deviation (SD).

The initial screening assay was repeated twice and the hits confirmed by the determination of IC_50_ (HTRF) in quadruplicates. IC_50_ (HTRF) was defined as the compound concentration at which the combination of ACE2 and S-RBD decreased by 50%.

### Pseudovirus neutralization assay on ACE2-overexpressing 293T cells

To further test the inhibiting effect of the candidate compounds on the binding of S protein and ACE2 at the cellular level, we performed pseudovirus neutralization assay [4, 5]. One day before SARS-CoV-2 pseudovirus transduction (day 0), 293T cells were washed once with D-PBS and dissociated using 0.25% of Trypsin. Approximately 3 × 10^4^ ACE2-overexpressed and vector-overexpressed 293T cells were seeded in each well of the 96-well plates at 37 °C with 5% CO_2_ overnight. On the first day of SARS-CoV-2 pseudovirus transfection (day 1), the frozen SARS-CoV-2_S (D614G)-pseudotyped lentivirus was melted on ice and gently pipetted several times to mix the dissolved virus particles. Then, 50 μl of virus solution was added to 450 μl of fresh complete culture medium (DMEM + 10% FBS) containing 5 μg/ml of polybrene, and mixed gently. And the candidate compounds TS-984, TS-1276 and nafamostat mesylate were made into 100 mM of stock solutions with DMSO, meanwhile, TA was prepared in 100 mM of stock solutions with PBS. Then all the stock solutions were diluted to 50 and 100 μM with the mixture of fresh complete culture medium with virus. TA was diluted to 15 and 30 μM with the mixture of fresh complete culture medium with virus. The original medium was then changed with 70 µl of the above mixture with candidate compounds. Finally, the plate was shaken gently so that the virus solution covered every cell, and then placed into a carbon dioxide incubator at 37 °C and 5% CO_2_ for culturing. After 24 h infection, the cultures were subjected to fluorescence measurement using a Nikon ECLIPSE Ti2.

### Pseudovirus neutralization assay on ACE2-overexpressing pancreatic carcinoma cell line Capan2

One day before SARS-CoV-2 pseudovirus transduction (day 0), Capan2 cells were washed once with D-PBS and dissociated using 0.25% of Trypsin. Approximately 3 × 10^4^ ACE2-overexpressed and vector-overexpressed Capan2 cells were seeded in each well of the 96-well plates at 37 °C with 5% CO_2_ overnight. On the first day of SARS-CoV-2 pseudovirus transfection (day 1), the frozen SARS-CoV-2_S (D614G)-pseudotyped lentivirus was melted on ice and gently pipetted several times to mix the dissolved virus particles. Then, 50 μl of virus solution was added to 450 μl of fresh complete culture medium (DMEM + 10% FBS) containing 5 μg/ml of polybrene, and mixed gently. And the candidate compounds TS-984, TS-1276 and nafamostat mesylate were made into 100 mM of stock solutions with DMSO, meanwhile, TA was prepared in 100 mM of stock solutions with PBS. Then TS-984, TS-1276, and nafamostat mesylate were diluted to 50 and 100 μM with the mixture of fresh complete culture medium with virus. TA was diluted to 15 and 30 μM with the mixture of fresh complete culture medium with virus. The original medium was then changed with 70 µl of the above mixture with candidate compounds. The original medium was then changed with 70 µl of the above mixture and a certain volume of concentration-graded candidate compounds. Finally, the plate was shaken gently so that the virus solution covered every cell, and then placed into a carbon dioxide incubator at 37 °C and 5% CO_2_ for culturing. After 24 h infection, the cultures were subjected to fluorescence measurement using a Nikon ECLIPSE Ti2.

### Molecular docking

HTRF-based assay and pseudovirus neutralization assay suggested that TS-984 effectively inhibited the binding of the coronavirus S-protein and the human cell AEC2 receptor. To understand the structural basis of the inhibitory effects, we further investigated the binding mode of TS-984 to ACE2. The docking of TS-984 and the S protein/ACE2 complex was completed with the software Autodock 4.0 [6]. Firstly, the 2D structures of TS-984 were constructed in chimera [7] and optimized in autodock 4.0. There are 10 different conformations for the ligand TS-984. The crystal structure of the S protein/ACE2 complex was obtained from PDB database and the protein ID was 6m0j [8]. The simple optimization to the S protein/ACE2 complex includes adding the side chain of amino acid residues, adding the missing loop part in the crystal structure, distributing the protonation state of amino acid residues, and optimizing the whole protein structure under the condition of OPLS2005 [9] force field. In the docking process, 500 positions are generated in the initial stage, and the highest scoring-100 positions are minimized by conjugate gradient minimization. Q-site was used to find the possible binding pocket of TS-984 in the S protein/ACE2 complex structure.

### Statistical analyses

Data are presented as means ± SD. Student’s t tests were performed for all the experiments, except where indicated differently in the figure legends.

## Results

### Optimization of HTRF assay for high-throughput screening

**Fig. 1:**
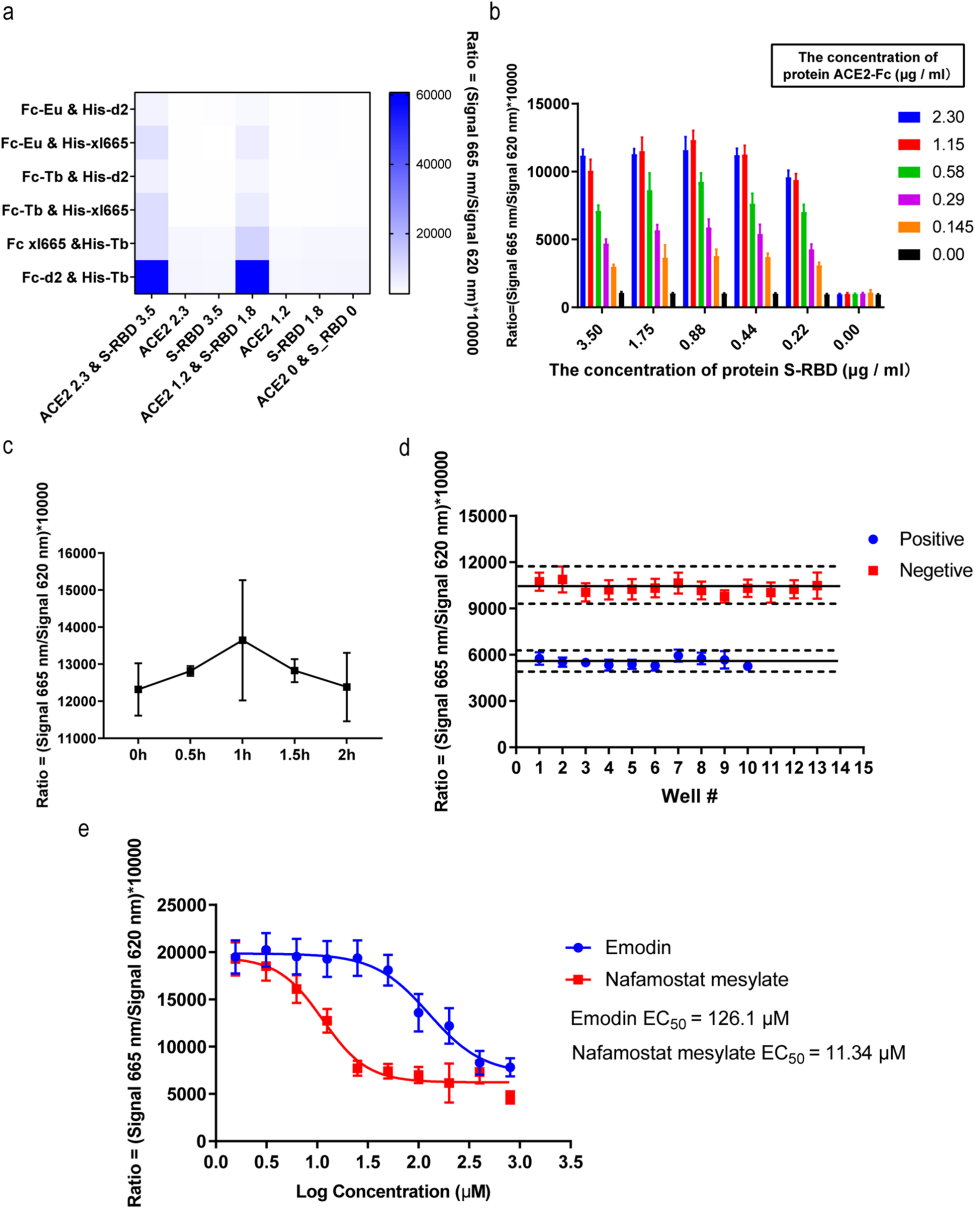
The establishment of the HTRF high thought put screening system based on the combination of ACE2 and S-RBD. **A** The selection of the tag antibody. The Fc-d2 and His-Tb pair can lead to the highest signal ratio at the same concentration of ACE and S-RBD. **B** The optimization of substrate concentration. The combination of ACE2-Fc at 1.15 μg/ml and S-RBD at 0.88 μg/ml can reach the highest signal ratio. **C** There is no significant change of the signal with the time. **D** Nafamostat mesylate was selected as positive control. The EC_50_ of Nafamostat mesylate was 11.34 μM. **E** The verification of the high through put system show that the Z factor was 0.67 which was good enough for the high though put screening

To obtain the maximal binding effect of the coronavirus S protein and its ACE2 receptor, we first optimized the ratios of S-RBD/ACE2 and S-RBD-His/ACE2-d2 (Fig. 1A–D). We observed that the assay system worked the best with 1.15 μg/ml of ACE2-d2 and 0.88 μg/ml of S-RBD-His. To ensure the HTRF assay was suitable for high throughput screening of the S protein-ACE2 inhibitors, natural compound emodin was used as a positive control in this study as it was previously identified to block the binding of the coronavirus S protein to the ACE2 receptor [2]. PBS with 1% BSA was used as a negative control. The average Z factor value of the assay was 0.67 (Z > 0.4), indicating the HTRF assay was suitable for screening. The HTRF signal was expected to decrease correspondingly if the compound under testing exhibited the inhibition effect on the binding of S protein and ACE2.

### Nafamostat mesilate inhibits the binding of SARS-CoV-2 S-RBD to ACE2

Nafamostat mesilate was reported to inhibit TMPRSS2. To see whether it could also block the interaction of the coronavirus S protein and its ACE2 receptor, we tested its inhibiting potential with our HTRF high throughput screening platform. Our results indicate that nafamostat mesylate inhibits the interaction of the S protein and ACE2, and its inhibiting effect is more powerful compared with the positive control compound. The EC_50_ for nafamostat mesilate and positive control emodin were 11.34 μM and 126.1 μM, respectively (Fig. 1E).

### Novel inhibitors identified against the binding of SARS-CoV-2 S-RBD to ACE2

**Fig. 2:**
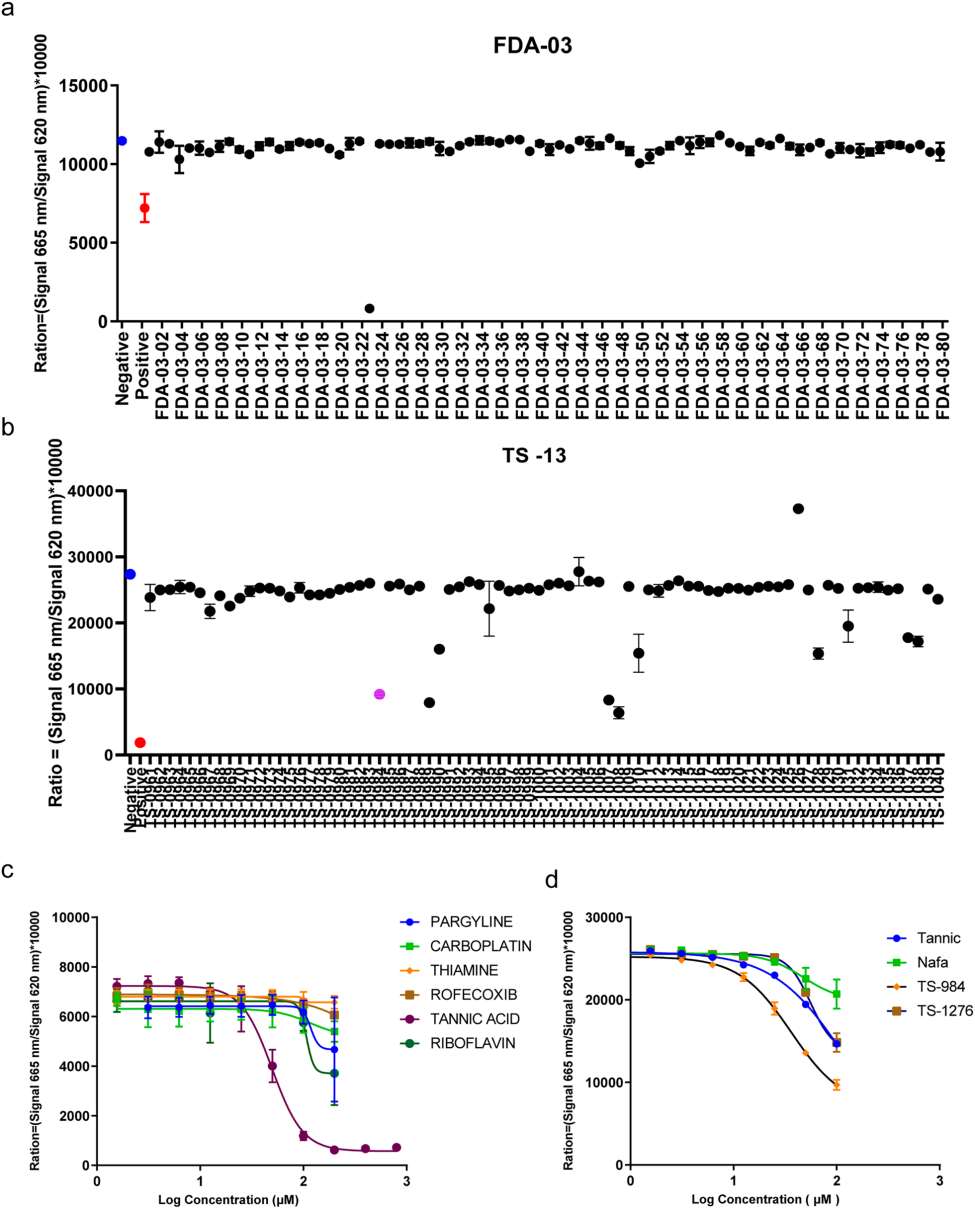
High through-put screening of the compound library. **A** The candidate compound FDA-03-23, tannic acid, can inhibit the combination of ACE2 and S-RBD greatly, was selected from 1280 kinds of compounds in FDA compound library. **B** TS984 (purple) is one of the compounds which can inhibit the combination of ACE2 and S-RBD. **C** The dose–effect curve of FDA-03-23. (EC_50_ = 49.71 µM). (**D**) The dose–effect curve of TS-984 (EC_50_ = 36.21 µM) and TS-1276 (EC_50_ = 16.38 µM)

To identify novel S-RBD/ACE2 binding inhibitors with our HTRF-based screening platform, we screened an FDA compound library of 1280 molecules and a Topscience compound library with 1584 natural products. The compound libraries were initially screened with our high throughput HTRF platform by using a concentration of 100 μM of each compound (Fig. 2A, B). In the initial screening, we identified 23 candidate compounds that presented an inhibition effect on the interaction between the SARS-CoV-2 S-RBD and ACE2, with an HTRF inhibition signal > 50%. Of the 23 compounds, 20 were excluded in the following validation experiments. Finally, only three compounds, tannic acid from the FDA library, TS984 (9-Methoxycanthin-6-one) and TS1276 (anthraquinone) from the Topscience library passed the EC_50_ (HTRF) determination by the HTRF screening. The EC_50_ (HTRF) for tannic acid, 9-Methoxycanthin-6-one, and anthraquinone was 49.71 μM, 36.21 μM, and 55.9 μM, respectively.

### TS984 effectively blocks pseudovirus entry into ACE2-overexpressing cells

**Fig. 3:**
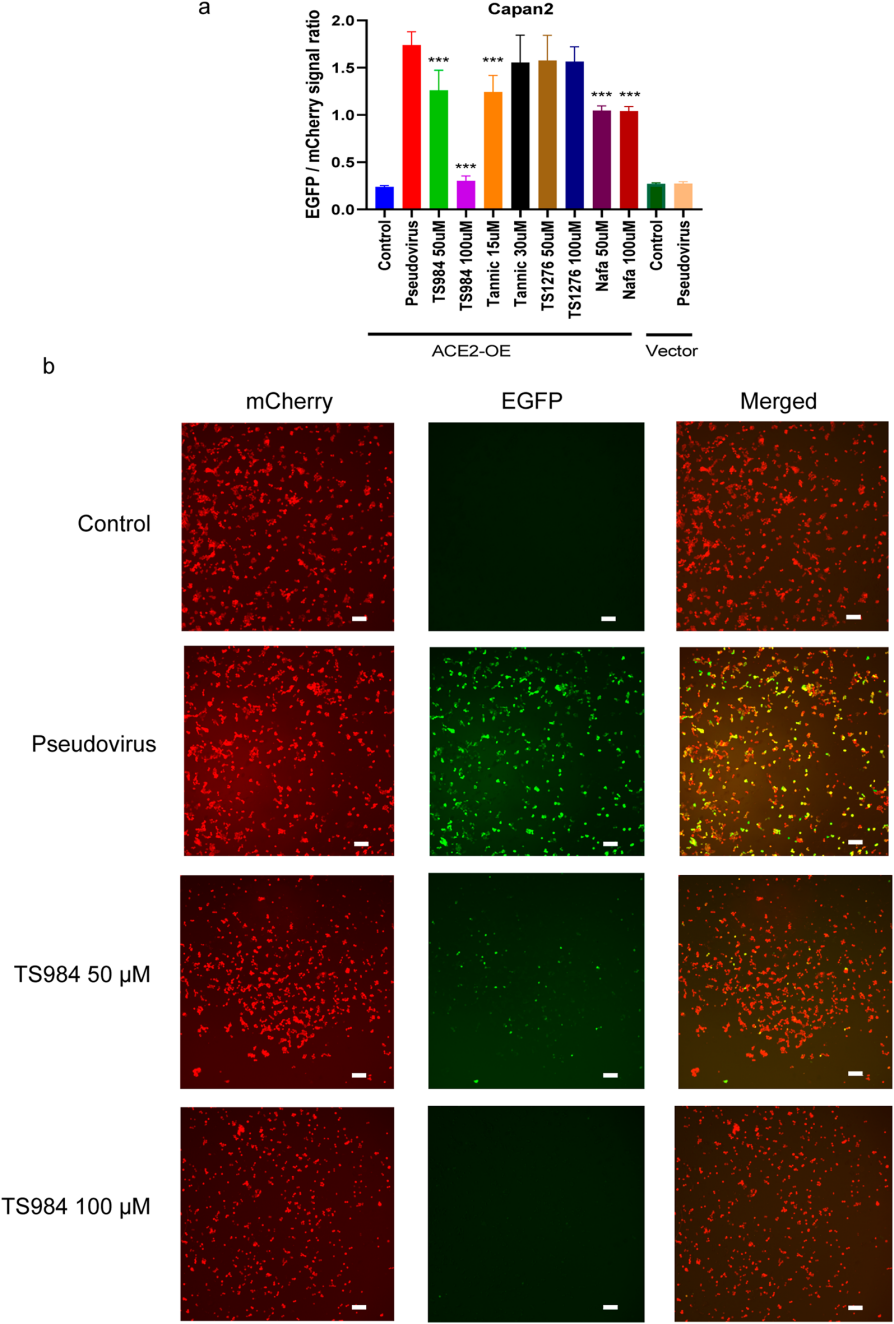
TS-984 can inhibit the SARS-COV-2 pseudo virus entering the Capan2 with ACE2 overexpression. **A** TS-984 can greatly reduce the EGFP/mCherry signal ratio. [*P < 0.05 and **P < 0.01 in comparison to control group]. **B** The 10× fluorescence image show that TS984 can inhibit the entering of pseudoviurs (green) into the Capan2 with ACE2 overexpression. (Scar bar, 200 μm)

**Fig. 4:**
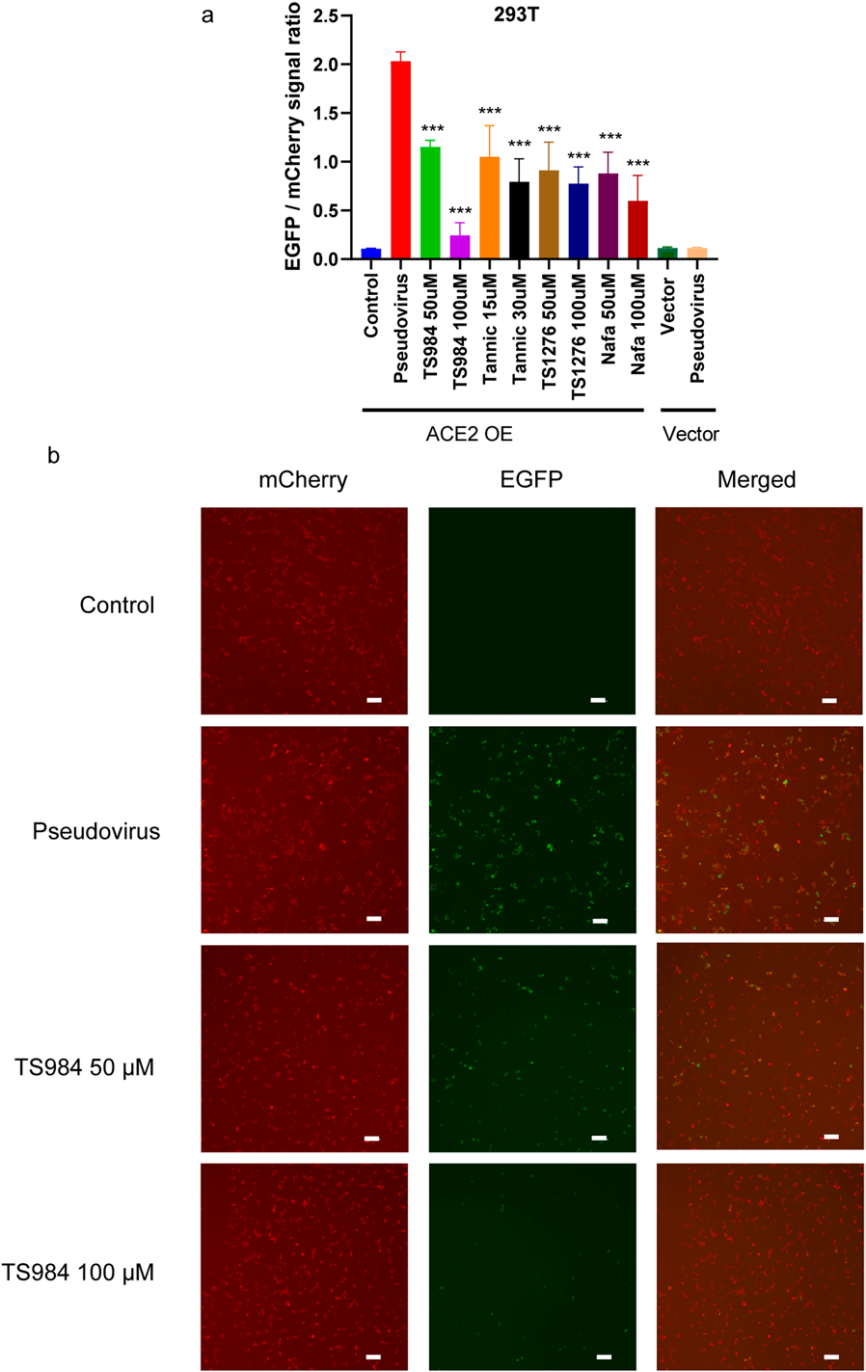
TS984 can inhibit the SARS-COV-2 pseudo virus entering the 293T with ACE2 overexpression. **A** TS984 can greatly reduce the EGFP/mCherry signal ratio. [*P < 0.05 and **P < 0.01 in comparison to control group]. **B** The 10× fluorescence image show that TS984 can inhibit the entering of pseudoviurs (green) into the 293T with ACE2 overexpression. (Scar bar, 200 μm)

For our pseudovirus neutralization assay with ACE2-expressing 293T cells, all candidate compounds (nafamostat mesylate, tannic acid, TS984, TS1276) showed significant inhibiting effects on the entry of the pseudovirus into the ACE2-expressing 293T cells (Fig. 3A, B, Additional file 1: Fig. S1, Additional file 2: Fig. S2). Cancer patients are more susceptible to SARS-CoV-2 infection. ACE2 expression was elevated in pancreatic adenocarcinoma. We then tested these four compounds on ACE2-overexpressing Capan2 cells [10]. Of the compounds, TS984 presented the strongest inhibiting effect. In contrast, when Capan2 cells were used for our pseudovirus neutralization assay, nafamostat mesylate, tannic acid, and TS984 exhibited an inhibiting effect on the entry of the pseudovirus into ACE2-overexpressing Capan2 cells (Fig. 4A, B). Similarly, the inhibiting effect of TS984 was the strongest and was dose-dependent while tannic acid presented only a mild inhibiting effect at a low concentration (15 µM). Since tannic acid exhibits strong cytotoxicity to cells at a high concentration (> 30 µM), we did not observe any significant inhibiting effect on the entry of the pseudovirus into Capan2 cells. TS1276 did not present any significant inhibiting effects either.

### Molecular docking with SARS-CoV-2 S-RBD

**Fig. 5:**
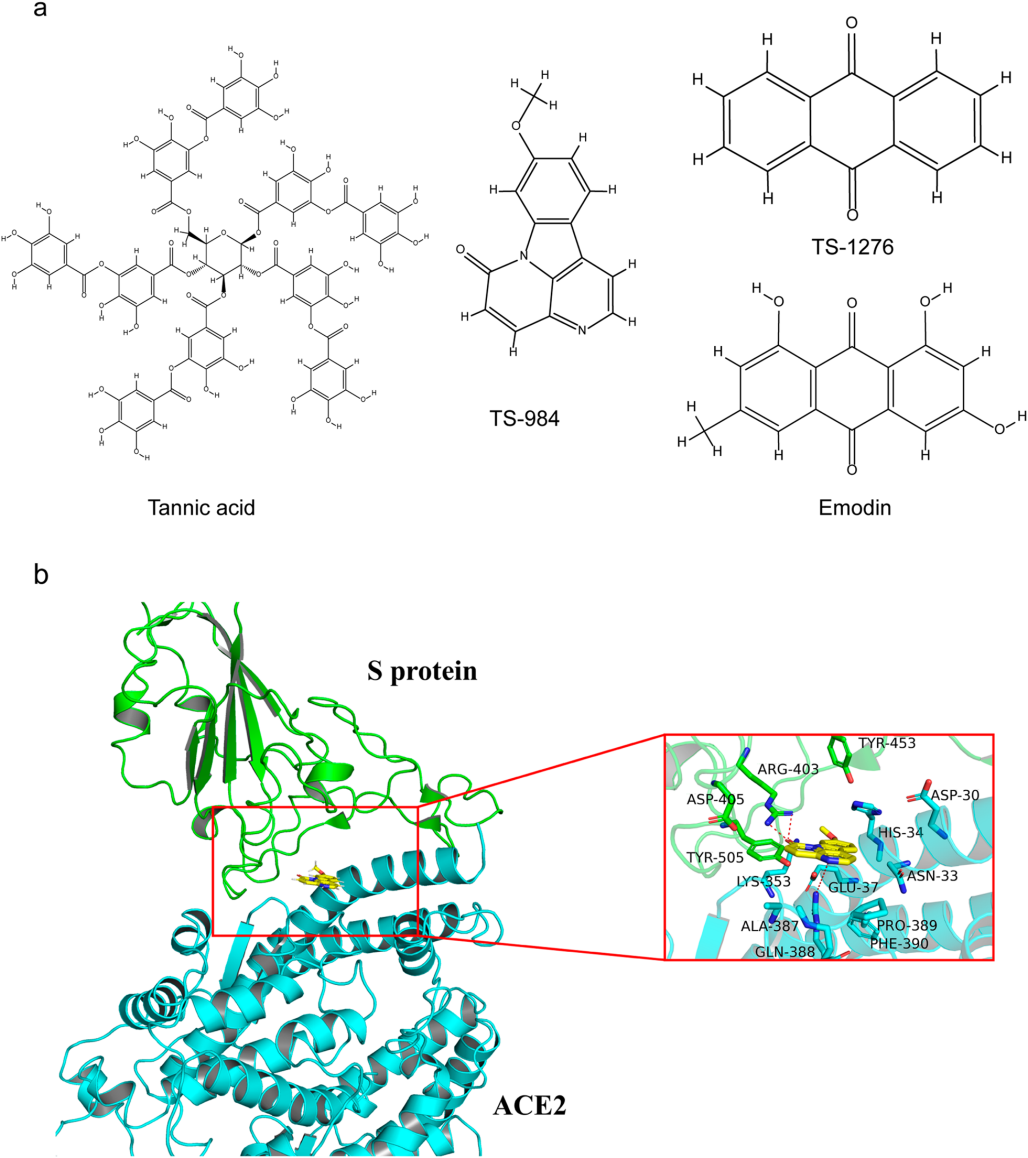
Molecular Docking of TS984 with S protein/ACE2 complex. **A** 2D Structure of Tannic acid, TS-984, TS-1276 and Emodin. **B** Predicting the interaction mechanism of the 9-methoxycanthin-6-one and the S protein/ACE2 complex. The yellow sticks represent the ligand 9-methoxycanthin-6-one, the green and blue sticks represent the important residues within 5 Å of the ligand, the red dotted line represents the H-bond interaction located in the 9-methoxycanthin-6-one and the S protein/ACE2 complex

Docking simulation indicates that and TS-984 is tightly “locked” in the binding pocket of ACE2 by establishing abundant hydrogen bonds with the surrounding residues. The occupation of TS-984 prohibits the binding of the S-protein to ACE2, subsequently, blocking the interaction between S-protein and ACE2 (Fig. 5). The predicted binding pocket at the interface between S protein and ACE2 protein was used to define the binding site, and then the ligand TS-984 was docked in the binding site (Fig. 5B). The residues involving in the interaction of TS-984 and the S protein/ACE2 complex include ARG403, ASP405, TYR453 and TYR505 in the S protein, and ASP30, ASN33, HIS34, GLU37, LYS353, ALA387, GLN388, PRO389, and PHE390 in ACE2. The interactions of these residues for the binding of TS-984 to the S protein/ACE2 complex are mainly polar (e.g., ASP30, HIS34, GLU37, LYS353, ALA387, GLN388, ARG403, ASP405, TYR453, and TYR505). In addition, TS-984 has hydrogen bonding with GLN388 and ARG403. All of these interactions play a critical role for maintaining the stability of TS-984 binding to the S protein/ACE2 complex.

## Discussion

Currently, there are few effective anti-SARS-CoV-2 drugs available for clinically treating COVID-19. Given the urgency of the pandemic, agencies and companies worldwide are giving top priority to repurpose existing drugs. Up to now, several small-molecule compounds have been repurposed for treating COVID-19 [11]. These compounds include Hydroxychloroquine (HCQ) developed by Sanofi, Remdesivir by Gilead, Favipiravir by Toyama, Lopinavir-ritonavir by Abbott, fluvoxamine by SolVay Pharmaceutics, molnupiravir by Merck and Ridgeback, and Paxlovid by Pfizer [11–13]. These agents are considered to exert their antiviral effects through different mechanisms such as blocking viral entry into host cells, inhibiting an essential virally encoded enzyme, targeting a host enzyme required for viral replication, and obstructing coronavirus particle formation [14]. However, while some early studies have reported that these agents appeared to inhibit SARS-CoV-2, large-scale clinical trials showed that most of them failed to provide significant benefits for hospitalized COVID-19 patients. For instance, Remdesivir, approved for use in patients with severe COVID-19, is believed to deter virus from replication by inhibiting a critical RNA polymerase, it was never demonstrated to effectively block SARS-CoV-2 infection and improve clinical outcomes [15–18]. Other large-scale clinical data have further revealed that Remdesivir does not exert any significant anti-SARS-CoV-2 effects in patients with severe COVID-19 [19, 20].

TMPRSS2, a serine protease, primes the S protein of human coronaviruses MERS-CoV and SARS-CoV, and promotes its entry into the host cell. Camostat mesilate, a protease inhibitor developed for the treatment of pancreatitis in Japan in the 1980s, was reported to inhibit TMPRSS2 and block the entry of SARS-CoV and SARS-CoV-2 into host cells [21]. Previous studies have demonstrated that camostat mesilate suppresses virus-cell membrane fusion in vitro and in vivo, thus, viral replication [22, 23]. Another study revealed that camostat mesilate blocks the entry of the SARS-CoV-2 virus into the lungs [24]. Therefore, camostat mesilate has been repurposed for the treatment of COVID-19 in clinical trials [25].

Nafamostat mesilate was also found to suppresses TMPRSS2 and virus-cell fusion, and thus block the entry of SARS-CoV-2 virus into host cells [26, 27]. Notely, nafamostat mesilate displayed a much more potent inhibiting effect compared with camostat mesilate on TMPRSS2 [23]. In present study, we further demonstrated that nafamostat mesilate can block the interaction of the coronavirus S protein and its host ACE2 receptor. Based on our results, nafamostat mesilate exerted a more effective inhibiting power on the binding of the S protein to ACE2 compared with the positive control emodin. Thus, nafamostat mesilate is a dual inhibitor of TMPRSS2 and the binding of the S protein to its ACE2 receptor.

Cellular entry of SARS-CoV-2 relies on two key procedures: the activation of the viral surface S protein by TMPRSS2 proteolytic processing, and the binding of the activated S protein (S1) to the cell surface receptor ACE2 for fusion of the virus-cell membrane. On the other side, maturation of the SARS-CoV-2 virions in host cells depends on a proteolysis of the viral precursor polyprotein by the main protease (M^pro^/3CL^pro^). A previous study uncovered that tannic acid is able to inhibit both TMPSS2 and the main protease (M^pro^/3CL^pro^). Thus, tannic acid is a potential dual inhibitor of both the SARS-CoV-2 main protease (M^pro^) and the TMPRSS2 [28]. Apparently, targeting the TMPRSS2 as well as M^pro^ is a better option for the treatment of COVID-19. However, no previous studies have investigated whether nafamostat mesilate or tanic acid can inhibit the binding of the S protein and ACE2 as well. To date, except for emodin which shows only a moderate inhibition effect [2], no other compounds have been reported to exert an inhibition effect on the binding of the S protein and ACE2. In this study, we demonstrated that tannic acid inhibits the binding of the S protein to the ACE2 host cells, indicating that tannic acid is a triple inhibitor for TMPRSS2, M^pro^, and S-ACE2 binding. At present, Tannic acid is the only identified drug that can inhibit TMPRSS2, M^pro^, and the interaction between the coronavirus S protein and the ACE2 human cell receptor. However, our data shows that tannic acid has higher cytotoxicity that leads to cell death when the effective concentration is applied. For this reason, tannic acid might be an unsuitable drug for SARS-CoV-2 treatment, as it cannot be directly repurposed for the treatment of COVID-19 patients before the cytotoxicity is reduced.

In this present study, we identified a more potent inhibitor for blocking the binding of the S protein and ACE2. TS984 (9-Methoxycanthin-6-one), is an indole alkaloid and one of the main constituents in Eurycoma longifolia and Simaba multiflora. TS-984 has never been used as an antineoplastic and antiplasmodial agent [29–31]. In this study, we identified for the first time that TS984 is able to block the binding of the coronavirus S protein to the host ACE2 receptor. Compared with tannic acid and nafamostat mesilate, TS984 (9-Methoxycanthin-6-one) presented a much stronger inhibiting effect with a lower cytotoxicity. We also demonstrated that TS-1276, anthraquinone, exhibited an inhibiting effect on the binding of the S protein to ACE2. But the effect of TS-1276 was much weaker compared with TS-984. Intriguingly, both TS-1276 and emodin belong to anthraquinone compounds. Emodin is a derivative of TS-1276.

## Conclusions

In summary, while emodin, and TS1276 are moderate inhibitors, nafamostat mesilate and tannic acid exhibit stronger inhibiting effects on the interaction of the S protein and ACE2. Furthermore, nafamostat mesilate is a dual inhibitor and tannic acid is a triple blocker for SARS-CoV-2 infection. In this present study, we demonstrated that TS984 is the most effective agent for blocking the binding of the coronavirus S protein to the host ACE2 receptor. TS984 appears to be a promising drug against SARS-CoV-2 and might have the potential to be repurposed for the treatment of COVID-19 patients.

## Supplementary Information

**Additional file 1: Figure S1.** TS-984 can block the entry of SARS-CoV-2 pseudovirus into Capan2 cells with ACE2 overexpression. Capan2 with ACE2 overexpression infected with pseudovirus under 40X microscope. TS-984 can effectively block the entry of pseudovirus into Capan2 cells with ACE2 overexpression in a dose-dependent manner. (Scar bar 100 μm).

**Additional file 2: Figure S2.** Tannic acid and Nafamostat mesylate block the pseudoviruses from entering Capan2 ACE2 overexpressing cells. Capan2 with ACE2 overexpression infected with pseudovirus under 10× microscope. (Scar bar 200 μm).

## Data Availability

Currently, TS-984 is under preclinical development toward IND (Investigational New Drug) filing. TS-984 is available from TOPSCIENCE under the name of MT4601. All data are available in the main text or the supplementary materials. All data have been uploaded onto the Research Data Deposit (www.researchdata.org.cn) with the approval number 2206070002.

## Abbreviations

SARS-CoV-2: Severe acute respiratory syndrome coronavirus 2
COVID-19: Coronavirus disease 2019
HTRF: Homogeneous Time Resolved Fluorescence
TA: Tannic acid
ACE2: Angiotensin-converting enzyme 2
PBS: Phosphate buffered saline
DMEM: Dulbecco’s modified eagle medium
FBS: Fetal Bovine Serum
TMPRSS2: Transmembrane Serine Protease 2
CM: Camostat mesylate
M^pro^: Main protease
3CL^pro^: The 3C-like protease
