# Supplementary figures and images for: Potential inhibitors for blocking the interaction of the coronavirus SARS-CoV-2 spike protein and its host cell receptor ACE2
Source: J Transl Med. 2022 Jul 14;20:314. doi: 10.1186/s12967-022-03501-9 (PMC9281089; doi:10.1186/s12967-022-03501-9)

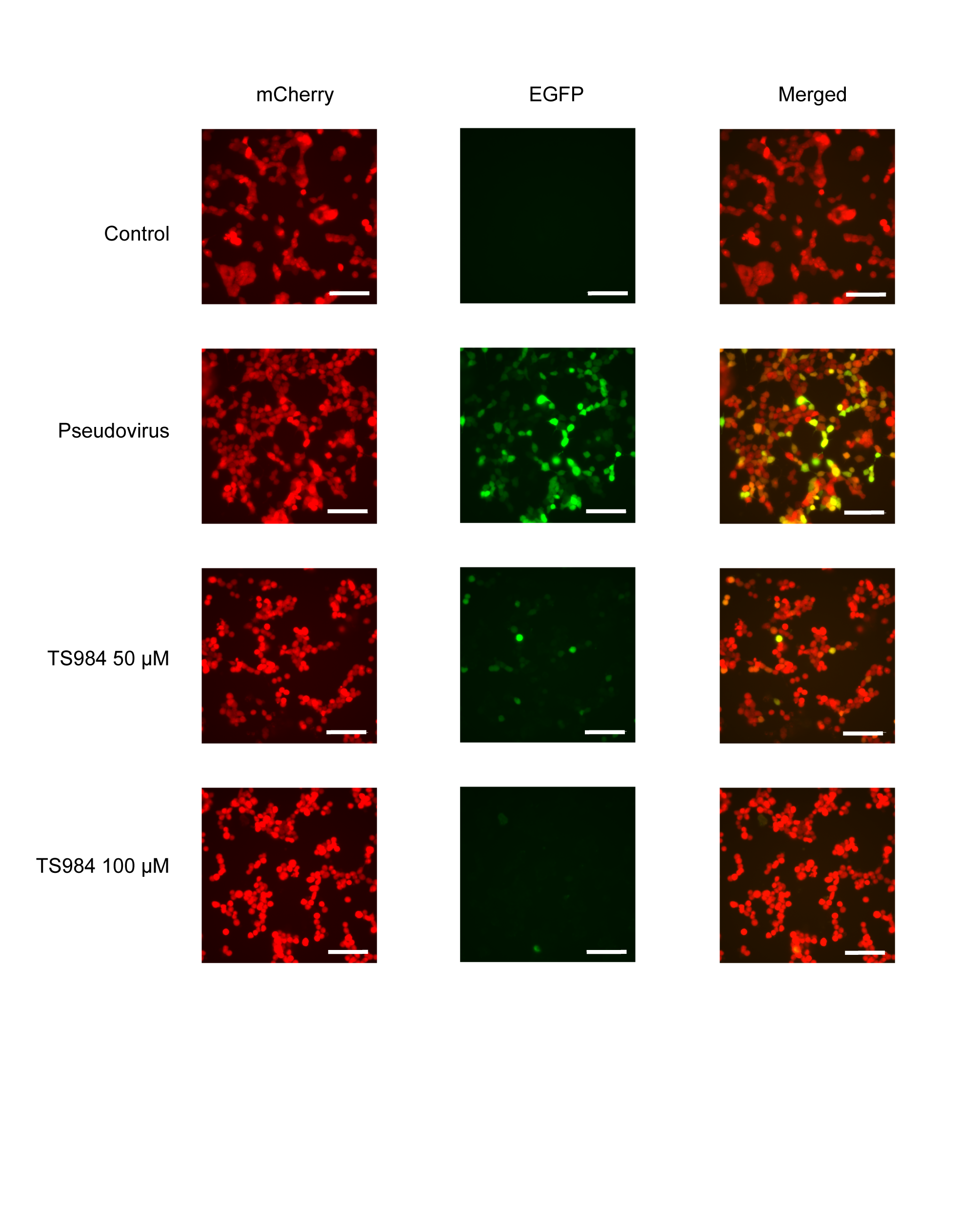

Supplement: Supplementary file 1 — Additional file 1: Figure S1. TS-984 can block the entry of SARS-CoV-2 pseudovirus into Capan2 cells with ACE2 overexpression. Capan2 with ACE2 overexpression infected with pseudovirus under 40X microscope. TS-984 can effectively block the entry of pseudovirus into Capan2 cells with ACE2 overexpression in a dose-dependent manner. (Scar bar 100 μm). [file 12967_2022_3501_MOESM1_ESM.tif]

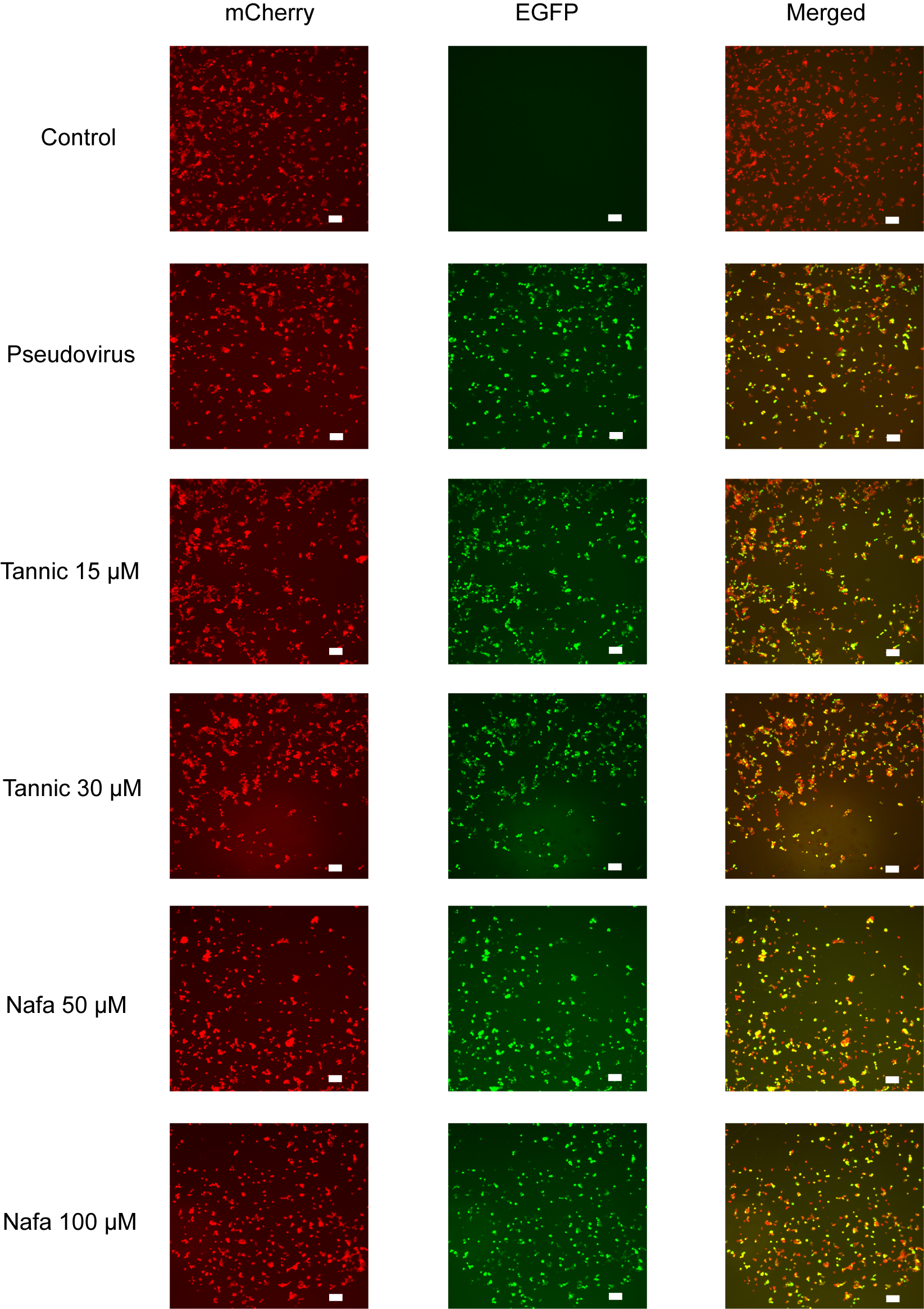

Supplement: Supplementary file 2 — Additional file 2: Figure S2. Tannic acid and Nafamostat mesylate block the pseudoviruses from entering Capan2 ACE2 overexpressing cells. Capan2 with ACE2 overexpression infected with pseudovirus under 10× microscope. (Scar bar 200 μm). [file 12967_2022_3501_MOESM2_ESM.tif]
